# Sesamin Protects the Femoral Head From Osteonecrosis by Inhibiting ROS-Induced Osteoblast Apoptosis in Rat Model

**DOI:** 10.3389/fphys.2018.01787

**Published:** 2018-12-11

**Authors:** Shuang Deng, Jian-Lin Zhou, Hong-Song Fang, Zhi-Gang Nie, Sen Chen, Hao Peng

**Affiliations:** Department of Orthopedics, Renmin Hospital of Wuhan University, Wuhan, China

**Keywords:** sesamin, ONFH, dexamethasone, ROS, apoptosis

## Abstract

Glucocorticoids intake has become the most common pathogenic factor for osteonecrosis of the femoral head (ONFH). Annually, tens of millions of patients suffer from pain related to ONFH. Researchers have proposed several underlying mechanisms of ONFH, including osteocyte apoptosis, cell differentiation disorder, and angiogenesis hindrance. Sesamin, isolated from *Sesamum indicum* seeds, was reported could affect osteocyte inflammation and differentiation in osteoarthritis and osteoporosis. We investigated the underlying influence of sesamin on ONFH rat model. Fifteen male Sprague-Dawley rats were randomly divided into three groups. The ONFH model group only received the methylprednisolone (MPS) and lipopolysaccharide (LPS) injection to promote the development of ONFH. The sesamin treatment group was injected with sesamin, MPS, and LPS. The control group was untreated. Rats from above groups were sacrificed 4 weeks later. The effect of sesamin on ONFH rats was validated by H&E staining. TUNEL staining showed that femoral head necrosis was attenuated by sesamin. Furthermore, the phosphorylation of Akt was increased and the downstream cellular apoptosis signal pathway was inhibited. Intracellular ROS level was decreased after sesamin treatment. In conclusion, our findings suggest that sesamin protects the femoral head from osteonecrosis by inhibiting ROS-induced osteoblast apoptosis.

## Introduction

Approximately 20,000–30,000 patients in the United States are diagnosed as ONFH annually (Moya-Angeler et al., 2015). Most patients are young individuals between 20 and 40 years of age, who will suffer lifelong symptoms (Larson et al., 2018). ONFH is divided into two categories: traumatic and nontraumatic. Chronic glucocorticoid (GC) steroid administration, alcohol abuse, and virus infection are thought to be the leading causes of nontraumatic ONFH. Of these, the cumulative intake of glucocorticoids is the most common pathogenic factor: 9–40% of patients receiving long-term glucocorticoid therapy develop ONFH (Weinstein, 2012; Xu et al., 2018).

Many investigations into the ONFH pathogenic mechanisms have been reported. To date, vasoconstriction, apoptosis, oxidative stress, lipid metabolism disturbances, and disturbances of the coagulation-fibrinolysis system are thought to be the molecular mechanisms of ONFH (Zheng et al., 2014; Yang et al., 2016). The interaction of glucocorticoid receptor-related kinases and phosphatases alters inflammation in GC-induced animal models, leading to side effects including ONFH (Beck et al., 2009). In murine osteoblastic MC3T3-E1 cells, proliferation was sustained at the G1 phase as a result of Dex stimulation, which activated cellular apoptosis (Li et al., 2012). The elevation of ROS expression resulted in endoplasmic reticulum (ER) stress, and triggered the apoptosis of primary osteocytes in C57BL/6 mice (Sato et al., 2015). It is now widely accepted that ONFH is a multi-genesis disease, which requires the development of more animal models, more intensive study, and better therapeutic agents.

Originally identified in sesame (*Sesamum indicum*), sesamin is found in 30 different plants belonging to different genera, including *Sesamum*, *Virola*, *Piper*, *Camellia*, and *Magnolia* (Akl et al., 2013; Dou et al., 2018). Sesamin was reported to have various anti-cancer functions, including protecting cells from oxidative stress, reducing tumor cell proliferation, inhibiting inflammatory processes, and stimulating cellular apoptosis (Nakano et al., 2003; Hou et al., 2004; Fujikawa et al., 2005; Banjerdpongchai et al., 2010; Chung et al., 2010). Previous studies reported that sesamin suppressed tumor progress at the cellular level. In HepG2, a human hepatocellular carcinoma cell line, sesamin significantly inhibited proliferation by arresting cells at the G2/M phase, leading to the activation of STAT3 and the apoptosis pathway (Deng et al., 2013). In another study, researchers demonstrated that sesamin activated Bax, caspase-3, p53, and checkpoint kinase 2 (Siao et al., 2015). In contrast, cells were protected from inflammation in other studies. Sesamin attenuated inflammation in LPS-induced acute lung injury BALB/c mice by suppressing LPS-induced cytokine (TNF-α, IL-6, and IL-1β) production (Qiang et al., 2016). In TAC-induced cardiac hypertrophy mouse models, sesamin suppressed hypertrophic and fibrotic responses via the Sirt3/ROS pathway (Fan et al., 2017).

The function of sesamin in osteoarthritis and osteoporosis has also been investigated. Sesamin inhibited IL-1β-stimulated inflammatory responses in primary osteoarthritis chondrocytes by activating the Nrf2 signaling pathway (Kong et al., 2016). In M-CSF- and RANKL-induced human PBMCs, sesamin had a significant inhibitory effect on osteoclast differentiation (Wanachewin et al., 2017). However, no studies have reported the influence of sesamin in the ONFH model.

In this study, we investigated the function of sesamin in an ONFH rat model and primary osteoblasts. H&E staining and TUNEL assay revealed that sesamin attenuated ONFH progression. Furthermore, experiments using primary osteoblasts indicated that Dex activated cellular apoptosis, which was reversed by sesamin, possibly by decreasing ROS expression. Our study is the first functional investigation of sesamin in ONFH, and provides a wider perspective for disease treatment.

## Materials and Methods

### Animals

This study was carried out in accordance with the recommendations of the Institutional Animal Care and Use Committee of Wuhan University, followed by the guidance for the Care and Use of Laboratory Animals (1996). The protocol was approved by the Institutional Animal Care and Use Committee of Wuhan University [No. WDRM (FU) 20180212]. A total of 15 male Sprague-Dawley rats aging at 8–10-week-old, weighing 250 ± 20 g, were got from the Hubei Provincial Center for Disease Control and Prevention, Wuhan, China. The rats were housed under specific pathogen-free (SPF) conditions. Besides, they were provided access to conventional chow and tap water *ad libitum*. All surgery was operated under anesthesia, and all attempts were made to minimize the suffering.

### Cell Culture

Primary osteoblasts were obtained from neonatal rats. All procedures were performed under sterile conditions. Briefly, rats were soaked in 75% ethanol. After washing with sodium phosphate buffer (PBS, Hyclone), the skulls and periosteum were obtained. Blood vessels and connective tissues were removed. Then skulls were cut into pieces and transferred into a culture flask. Four milliliters of trypsin was added into the flask for 5 min. After digestion was completed, Ham’s F-12 Nutrient Mixture (Hyclone) containing 10% fetal bovine serum (FBS) (Gibco) was used to terminate the digestion. The skull pieces were incubated with 0.1% collagenase I solution for 30 min; then centrifuged to collect the osteoblasts, which were cultured in Ham’s F-12 Nutrient Mixture containing 10% FBS at 37°C with 5% CO_2_. One hour later, the flask was turned over and the osteoblasts were continuously incubated. Once primary culture cells reached confluence over the entire bottom of the flask, they were ready for passage. Third generation cells were used in these experiments.

First, 1 × 10^8^ primary osteoblasts were cultured in 25 cm dishes and different amounts of sesamin were added to the culture media for 24 h. Then 1 μM Dex (Sigma–Aldrich, St. Louis, MO, United States) was incubated with the cells for another 24 h. Then cells were collected for further experiments.

### Materials

Sesamin (catalog no. S171302, purity > 98%) was purchased from Aladdin Industrial Corporation (Shanghai, China), and was suspended in 0.5% carboxymethylcellulose solution for animal experiments. Its structure was documented in PubChem (no. 24899834). LPS (*Escherichia coli* serotype 055: B5) and Dex were purchased from Sigma-Aldrich (St. Louis, MO, United States). Methylprednisolone (MPS) was purchased from Pfizer Pharmaceutical, China. Primary antibodies used in this study, rabbit anti-Bcl-2 polyclonal antibody (ab196495), rabbit anti-Bax monoclonal antibody (ab32503), rabbit anti-Akt polyclonal antibody (ab8805), and rabbit anti-phospho-Akt polyclonal antibody (ab38449) were purchased from Abcam Biotechnology (Cambridge, MA, United States). Rabbit anti-caspase-3 polyclonal antibody and mouse anti-GAPDH monoclonal antibody (no. 60004), used as a loading control, were purchased from ProteinTech Biotechnology.

### Experimental Design

In animal experiments, all rats were randomly divided into three groups. (1) Sesamin treatment group (*n* = 5): rats were injected with sesamin, LPS, and MPS in the first week, and given 0.9% saline over the next 3 weeks. On days 1 and 2, rats were given an intraperitoneal injection of sesamin (100 mg/kg) 2 h before the intravenous injection of LPS (1.8 mg/kg) (Kong et al., 2009, 2015; Zhang et al., 2013; Qiang et al., 2016). On days 3–7, the animals were given 25 mg/kg MPS intramuscularly 2 h after the intraperitoneal injection of sesamin to promote the development of femoral head necrosis as described by Okazaki et al. (2009, 2012). (2) ONFH model group (*n* = 5): rats were given LPS and MPS in the same manner as the sesamin treatment group in the first week, and 0.9% saline was given in the same mode for another 3 weeks. (3) Control group (*n* = 5): all rats were given 0.9% saline during the 4 weeks.

The animals were sacrificed at the end of the fourth week with an overdose injection of pentobarbital sodium. Subsequently, bilateral femoral heads from each rat were excised. The right femoral head was fixed in 4% paraformaldehyde, and the left femoral head was put in liquid nitrogen immediately and stored at -80°C until analyzed.

### Hematoxylin and Eosin Staining

Femoral head tissues were excised and fixed in neutral buffered solution for 48 h [4% formaldehyde in 0.1 M sodium phosphate buffer (PBS), pH = 7.4], decalcified in 10% ethylenediaminetetraacetic acid (EDTA) for 4 weeks, dehydrated in graded alcohols, and finally embedded in paraffin. Paraffin (5 μm) sections were mounted on glass slides and processed by hematoxylin and eosin (H&E) staining for general morphological evaluation. Three sections were obtained from each of five animals per group. A representative image is shown for each group.

### TUNEL Assay

Apoptotic osteoblasts and osteocytes were detected using the terminal deoxynucleotidyl transferase-mediated dUTP nick end-labeling (TUNEL) assay, with an *in Situ* Cell Death Detection Kit (Roche Diagnostics, Mannheim, Germany), according to the manufacturer’s instructions. Briefly, following routine deparaffinization and treatment with H_2_O_2_ (3%), the sections were digested with proteinase K (20 μg/mL, pH 7.4, 12 min) at 25°C and incubated with the reaction mixture (1:40, 60 min) at 37°C. Incorporated fluorescein was detected with horseradish peroxidase following incubation for 30 min at 37°C and were subsequently dyed with 3,3′-diaminobenzidine (DAB). Brown nuclei were assessed as positive apoptotic cells.

### Western Blotting

Osteoblasts were processed by M-PER mammalian protein extraction reagent (Pierce, Rockford, IL, United States) and protease inhibitor cocktail set III (Calbiochem, Darmstadt, Germany) plus 5 mmol/L EDTA. Overall, 20 μg of protein was loaded and separated by 10% SDS–PAGE. Then, the separated proteins were transferred to a PVDF membrane and blocked with 5% non-fat milk in Tris-buffer saline containing 0.05% Tween-20 (TBST). The membranes were incubated with the corresponding primary antibodies for 1 h at room temperature or overnight at 4°C. Then, the membranes were washed three times and incubated with a horseradish peroxidase-conjugated secondary antibody (1:10,000) in TBST for 1 h at room temperature. All specific bands were visualized by an ECL system kit (Pierce Biotechnology, Beijing, China) and the numerical value of the density was detected by ImageJ software (NIH, Bethesda, MD, United States). Additionally, densitometric data from each blot were standardized to the control conditions with the value set to one for each experiment and all tests were repeated three times.

### Quantitative Reverse Transcription PCR (qRT-PCR)

Total RNAs in primary osteoblasts were isolated using Trizol reagent (Invitrogen, Carlsbad, CA, United States) according to the manufacturer’s protocol. One microgram of extracted total RNA was used for reverse transcription. The M-MLV Reverse Transcriptase and RNas in Ribonuclease Inhibitor were purchased from Promega. Primers in this experiment were listed in Table 1. The targeted mRNA amount was measured by real-time PCR with GAPDH as an endogenous control. All experiments were repeated at least three times.

**Table 1:** Real-time quantitative PCR primer sequences.

Gene	Sequences
Bax	Forward: 5′-GGCGATGAACTGGACAACAA-3′
	Reverse: 5′-CAAAGTAGAAAAGGGCAACC-3′
Bcl-2	Forward: 5′-GGTGAACTGGGGGAGGATTG-3′
	Reverse: 5′-GCATGCTGGGGCCATATAGT-3′
Caspase-3	Forward: 5′-GGACCTGTGGACCTGAAAAA-3′
	Reverse: 5′-GCATGCCATATCATCGTCAG-3′
GAPDH	Forward: 5′-GAAGGTGAAGGTCGGAGTC-3′
	Reverse: 5′-GAAGATGGTGATGGGATTTC-3′


### Measurement of Intracellular ROS Levels

Changes in the ROS level in primary osteoblasts were detected using 2′,7′-dichlorofluorescein-diacetate (DCFH-DA, Sigma–Aldrich, St. Louis, MO, United States) staining. Cells were seeded into a 24-well plate with 3 × 10^3^ cells per well and exposed to different treatments. Then, adherent and floating cells in each group were harvested, washed with PBS three times, and stained with 10 μM DCFH-DA for 30 min at 37°C in the dark. The mean fluorescence intensity of each group was analyzed by flow cytometry (Bender MedSystems, Burlingame, CA, United States), which represented the intracellular ROS level.

### Statistical Analysis

In this study, all values were expressed as the mean ± standard deviation (SD) of at least three independent experiments. Statistical analyses were performed using SPSS 17.0 software (SPSS Inc., Chicago, IL, United States). The Student’s *t*-test was used for analyses and statistical significance was defined as *p* < 0.05.

## Results

### Sesamin Attenuates Osteonecrosis in SD Rats

**FIGURE 1:**
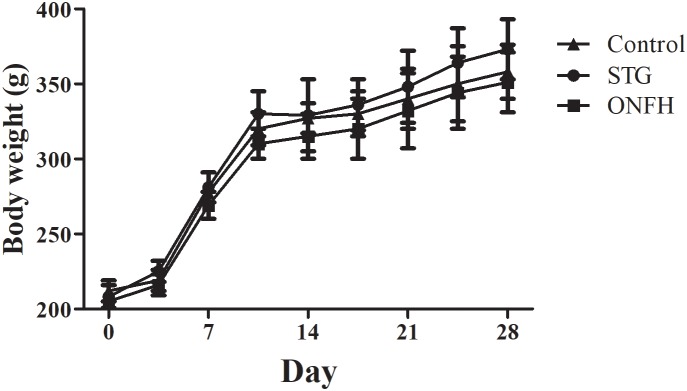
Body weight over time of rats in the sesamin treatment group, ONFH model group, and control group. Five rats were used in each group. STG, sesamin treatment group; ONFH, ONFH model group; Control, control group. Data values are expressed as mean ± SD.

To evaluate the influence of sesamin on ONFH rats, 100 mg/kg sesamin was intraperitoneally injected into normal rats, and then LPS and MPS were used to induce ONFH. The dose of sesamin used in this study was based on previous studies (Ma et al., 2015; Qiang et al., 2016). Throughout the study, no significant side effects were induced by sesamin. The mean body weight was not significantly different between the groups (Figure 1), and no accidental death occurred during the whole study. When injected with LPS and MPS, the femoral head of rats developed severe osteonecrosis. However, sesamin appeared to relieve pain in ONFH rats as determined by behavioral observation (data not shown).

The necrosis of femoral head was determined by macroscopic morphology observation. In normal group, bilateral femoral head was spherical and not collapsed. The surface of cartilage was ruddy and glossy, and smooth with homogeneous thickness. While in ONFH model group, the surface of cartilage became dim in color and lack of gloss. It was rough with minimal fibrillation or a slight yellowish discoloration. Different degrees of wear occur in some area. Since the treatment of Dex lasted for only 4 weeks, no femoral head collapse or osteophyte formation was found in the necrotic femoral head.

As in our experiment, no necrotic feature was observed in any rat or femoral head in control group. In the ONFH model group, necrosis was found in all five rats. Four out of the five rats had bilateral necrosis femoral head and the other one had unilateral necrosis femoral head (the rate of necrosis was 9/10). While in sesamin treatment group, necrosis was found in four rats. Only two out of the five rats had bilateral necrosis femoral head and another two had unilateral necrosis femoral head (the rate of necrosis is 6/10).

**FIGURE 2:**
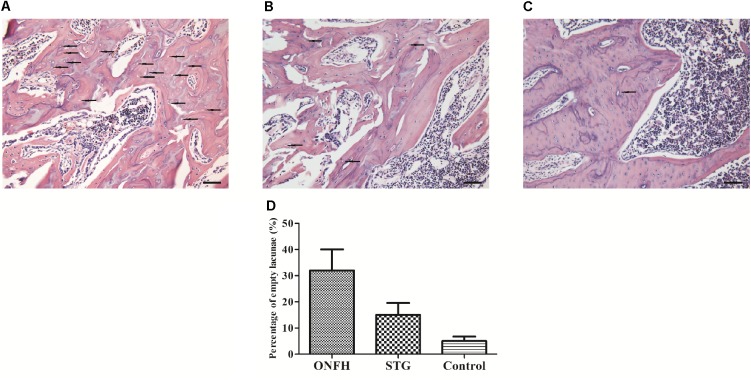
Histological appearance of the femoral head in three groups. **(A)** ONFH model group. **(B)** Sesamin treatment group. **(C)** Control group. The arrows indicated the empty lacunae. The original magnification of HE staining is 200×, and the scale bars were 100 μm. **(D)** The rate of empty lacunae in three groups. STG, sesamin treatment group; ONFH, ONFH model group; Control, control group. Data values are expressed as mean ± SD.

Microscopic morphology tests were used to validate the effect of sesamin (Figure 2). H&E staining was used to assess the general morphology of samples. In the ONFH model group (Figure 2A), the bone trabeculae showed pyknotic nuclei and empty lacunae, and fibrous tissues had accumulated in the medullary space, which are characteristic of severe osteonecrosis. Bone trabeculae in the sesamin treatment group (Figure 2B) rarely had pyknotic nuclei and empty lacunae suggesting osteonecrosis was inhibited. Besides, we calculated the rate of empty lacunae (Figure 2D). The results were counted from five randomly selected high-power fields in one section from each rat. These results correlated with the behavioral observations.

### Sesamin Reduces Apoptosis in the Osteonecrosis Zone

**FIGURE 3:**
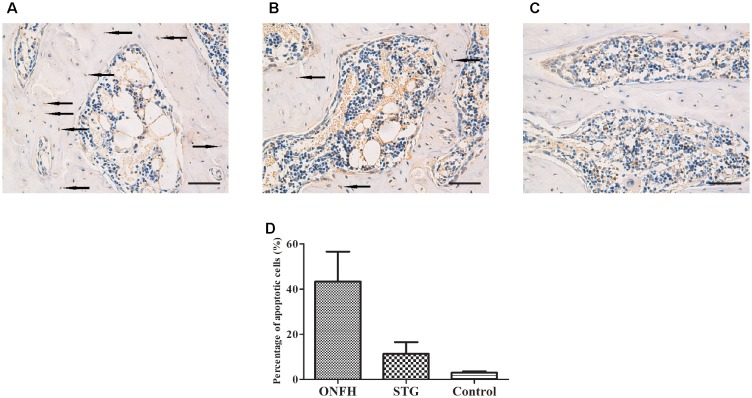
Photomicrograph of TUNEL staining showing evidence of apoptotic cells in the necrotic zone. **(A)** ONFH model group. **(B)** Sesamin treatment group. **(C)** Control group. The arrows indicated the apoptotic cells. The original magnification of TUNEL staining is 200×, and the scale bars were 100 μm. **(D)** The percentage of apoptotic cells in three groups. STG, sesamin treatment group; ONFH, ONFH model group; Control, control group. Data values are expressed as mean ± SD.

To determine whether the administration of sesamin affected apoptosis in the trabecular bone of the femoral head, we conducted TUNEL assays. We found that positively stained cells were detected in the ON zones in the ONFH model group and sesamin treated group (Figures 3A,B). However, the number of TUNEL-positive cells in high-power field was significantly reduced compared with that in ONFH model group (Figure 3C). Then we calculated the percentage of apoptotic cells in three groups (Figure 3D). The results were counted from five randomly selected high-power fields in one section from each rat. These data indicated that the administration of sesamin protected the trabecular bone cells from apoptosis.

### Osteoblast Apoptosis Is Inhibited by Sesamin

Prior to the analysis of the effects of sesamin on primary osteoblasts, we investigated the potential for cytotoxicity. Previously, sesamin at various concentrations (1.75–28 μM) and processing time (24, 48, and 72 h) were tested in a human fetal osteoblast cell line and showed that sesamin was not cytotoxic (Wanachewin et al., 2012). A similar result was obtained in another work using primary human articular chondrocytes (Phitak et al., 2012; Pothacharoen et al., 2014; Kong et al., 2016). In our study, cells were treated with 5, 10, and 20 μM sesamin for 48 h. No obvious cytotoxic effects were detected by MTT assay (data not shown).

**FIGURE 4:**
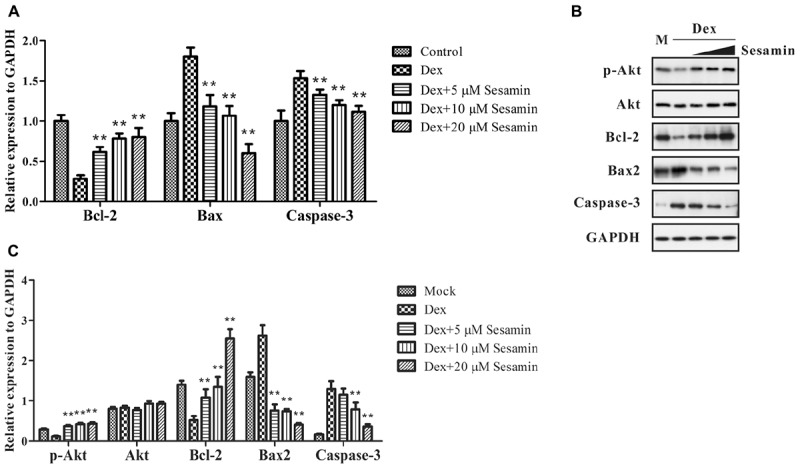
mRNA and protein expression or phosphorylation changes in Dex-induced apoptosis. The expression levels of phospho-Akt, Akt, Bcl-2, Bax, and caspase-3 were detected by mRNA **(A)** or western blotting **(B)** at 48 h after treatment. **(C)** Quantitative result of western blotting. All bands were measured for three times and normalized to GAPDH. Data values are expressed as mean ± SD. GADPH was utilized as endogenous control or to prove equal amounts of protein loading in each lane. M: Mock. ^∗∗^*p* < 0.05 vs. Dex treatment group.

In the Dex treatment group, 1 × 10^8^ primary osteoblasts were incubated with 1 μM Dex for 24 h. For the sesamin treatment group, 5, 10, or 20 μM sesamin was added into culture media at 24 h before Dex incubation, and cells were stimulated for another 24 h. The control group did not receive any treatment. During Dex-induced apoptosis, the proportion of Bax/Bcl-2 was increased, and caspase-3 was cleaved to induce apoptosis. Akt is upstream of Bax/Bcl-2, and the phosphorylation of Akt promotes the inhibition of apoptosis. Therefore, we detected the intracellular amounts of these proteins (Figure 4). The mRNA expression levels of Bax, Bcl-2, and caspase-3 were detected by qRT-PCR (Figure 4A). The results showed that Bcl-2 was upregulated when the concentration of sesamin was increased, while Bax and caspase-3 were downregulated in this process. Western blotting revealed that the phosphorylation of Akt was significantly upregulated in the sesamin treatment group compared with the Dex treatment group indicating that cellular apoptosis was inhibited (Figure 4B). Quantitative results of western blotting were measured in Figure 4C. This was confirmed by direct evidence that Bax and caspase-3 were decreased while Bcl-2 was increased. All the regulatory effects were dose dependent. Therefore, sesamin inhibits Dex-induced osteoblast apoptosis.

### Sesamin Reduces ROS Levels in Osteoblast

**FIGURE 5:**
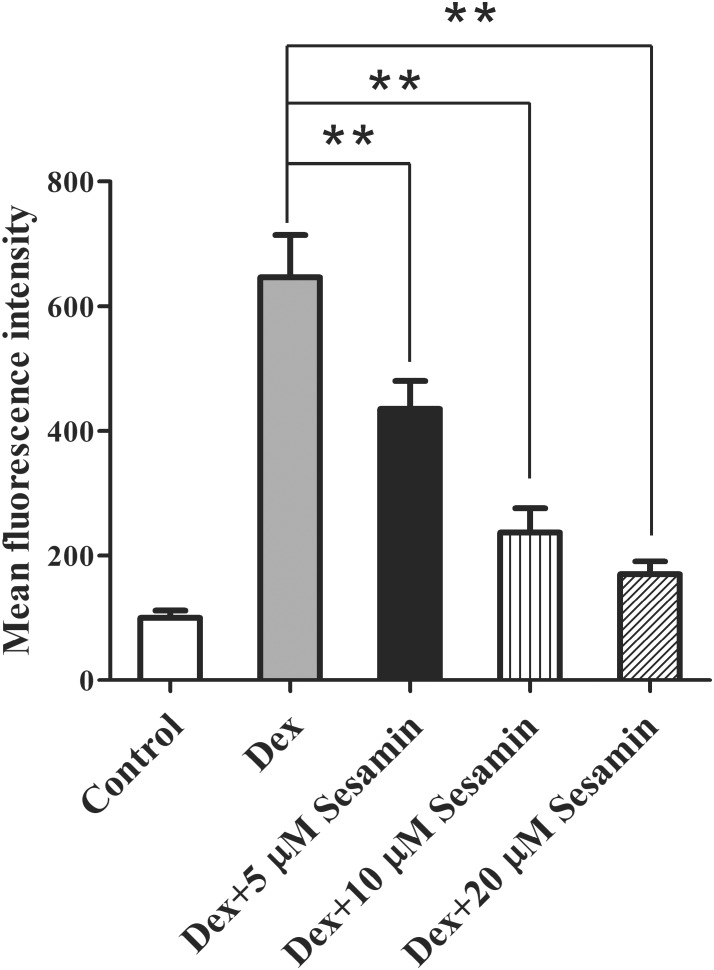
Downregulation of ROS expression by sesamin in Dex-treated osteoblast. DCFH-DA staining was used to determine the ROS levels in osteoblasts after sesamin and Dex treatment. The relative mean fluorescence intensities were presented as mean ± standard deviation (SD). ^∗∗^*p* < 0.01.

A previous work study suggested ROS inhibits the PI3K/Akt pathway and induces osteoblast apoptosis after Dex treatment (Lu et al., 2008; Li et al., 2015; Sato et al., 2015). Therefore, we detected the intracellular ROS level using DCFH-DA staining. 1 × 10^8^ primary osteoblasts were incubated with different doses (5, 10, and 20 μM) of sesamin for 24 h. After treatment with 1 μM Dex for another 24 h, osteoblasts were harvested. As shown in Figure 5, Dex increased ROS expression, but this effect was largely counteracted by sesamin, indicating that Dex-induced ROS expression was downregulated by sesamin.

## Discussion

Osteonecrosis of the femoral head has become a common disease with a high disability rate. Recently, there has been significant progression in diagnosis and therapeutics, yet its pathogenesis is still poorly understood. Among various hypotheses, apoptosis is one of the most studied mechanisms. Apoptotic signals transduced through the PI3K/Akt-Bax/Bcl-2/caspase 3 pathway has been reported in some orthopedic diseases (Li et al., 2012, 2015; Sato et al., 2015). In our study, the protective function of sesamin on glucocorticoid-treated femoral head and osteoblast was confirmed. H&E staining and TUNEL assay were used to demonstrate the influence of sesamin on glucocorticoid-induced ONFH. Akt-mediated apoptosis was inhibited and ROS expression was downregulated by sesamin. Taken together, we demonstrated the anti-apoptosis function of sesamin in femoral heads.

The accumulative administration of glucocorticoid accounts for dominant nontraumatic ONFH cases, but the pathogenesis of ONFH is still unknown. Noble et al. (1997) reported that osteocytes die by apoptosis. It is now widely accepted that the differentiation balance between osteoblasts and osteoclasts determines the fate of the femoral head (Weinstein, 2011, 2012; Jilka et al., 2013). Therefore, numerous studies have concentrated on osteoblast apoptosis. Phosphorylation activates Akt in osteoblasts to enhance the anti-apoptotic function of Bcl-2 (Ma et al., 2014). In our study, Akt was more active in the sesamin treatment group compared with the Dex treatment group. This suggests that sesamin affects cell apoptosis via the PI3K/Akt pathway.

A relationship between sesamin and apoptosis was demonstrated in breast cancer (Lee et al., 2011), hepatocellular carcinoma (Deng et al., 2013), cervical carcinoma (Dou et al., 2018), and leukemia (Banjerdpongchai et al., 2010). In contrast to our study, sesamin promoted cancer cell apoptosis to inhibit tumor genesis. This difference might be explained by the different cell types used in each study, because cancer cells show excessive proliferation. Sesamin contributes to the balance of cell differentiation and apoptosis. In cancer cells, sesamin decreases cell differentiation and proliferation, while in osteoblasts, apoptosis is inhibited to maintain the balance. This is supported by other reports in orthopedic diseases. In osteoporosis, sesamin promoted osteoblast differentiation in a human fetal osteoblast cell line via the MAPK signaling pathway (Wanachewin et al., 2012, 2017). Therefore, sesamin may positively regulate the MAPK signaling pathway in osteonecrosis; however, this requires further investigation.

The expression of ROS was elevated by Dex treatment. Excessive ROS may lead to cell apoptosis via the mitochondria-mediated caspase apoptosis pathway (Ravindran et al., 2011). In addition, ROS accumulation causes oxidative stress and activates the JNK pathway in osteoblasts, which might inactivate the Akt pathway and promote osteoblast apoptosis (Cakir et al., 2011). In our study, ROS was significantly downregulated in the sesamin treatment group compared with the Dex treatment group. This indicated that the inhibitory effect of sesamin on osteonecrosis was related to mitochondria. In a study by Ratana, the mitochondrial membrane potential was reduced dose-dependently in sesamin-treated U937 cells (Banjerdpongchai et al., 2010). However, whether sesamin acted as an anti-oxidant or pro-oxidant depended on the cell type and dose of sesamin. In our study, primary osteoblasts were used and the concentration of sesamin was 5–20 μM. This is a frequently used concentration in human fetal osteoblast cell lines and primary human articular chondrocytes (Phitak et al., 2012; Wanachewin et al., 2012; Pothacharoen et al., 2014; Kong et al., 2016). The results of our cellular experiment correlated with the ONFH rat model. Taken together, we suggest that sesamin is an antioxidant in osteonecrosis. Nevertheless, other cell lines and sesamin doses should be used to gain a better understanding of osteonecrosis.

Sesamin also regulates cell differentiation. It was reported that sesamin stimulated osteoblast differentiation (Wanachewin et al., 2012) and inhibited osteoclastogenesis (Wanachewin et al., 2017) via the p38/MAPK signaling pathway. Interestingly, the influence of sesamin on osteoblasts and osteoclasts is mediated via the phosphorylation regulation of p38 and ERK. Moreover, the concentration involved in both experiments was identical. Therefore, sesamin might be used in clinical therapeutics. Indeed, MAPK signaling regulates the fate of cells in many diseases. Li et al. (2016) demonstrated that sesamin inhibited the activation of p38/MAPK signaling in a human mast cell line to decrease mast cell-mediated allergic responses. In ischemic diseases, sesamin stimulated angiogenesis *in vitro* and *in vivo* through the activation of p38/MAPK pathways (Chung et al., 2010). Our study mainly studied the PI3K/Akt signaling pathway, which is involved in cellular apoptosis. Further studies should investigate the interactions between the PI3K/Akt and p38/MAPK pathways in osteonecrosis.

Glucocorticoid-induced ONFH has become a common public health problem. The effect of sesamin was reported in osteoarthritis and osteoporosis, but there were still no research in ONFH. In summary, our study reported a novel regulatory function of sesamin in osteonecrosis. We demonstrated that sesamin inhibited apoptosis in an ONFH rat model and primary osteoblasts, which may be related to ROS production. The findings suggest sesamin might be a therapeutic target for ONFH.

## Author Contributions

SD contributed conception and design of the study, finished most of the experiments, wrote the first draft of the manuscript, and completed the submission procedure. J-LZ, H-SF, and Z-GN helped with the animal experiments. SC performed the statistical analysis. All authors contributed to manuscript revision, read, and approved the submitted version.

## Conflict of Interest Statement

The authors declare that the research was conducted in the absence of any commercial or financial relationships that could be construed as a potential conflict of interest.

## Abbreviations

Dex: dexamethasone
LPS: lipopolysaccharide
ONFH: osteonecrosis of the femoral head
ROS: reactive oxygen species
